# Aortic Wall Elastic Properties in Case of Bicuspid Aortic Valve

**DOI:** 10.3389/fphys.2019.00299

**Published:** 2019-04-10

**Authors:** Guillaume Goudot, Tristan Mirault, Patrick Bruneval, Gilles Soulat, Mathieu Pernot, Emmanuel Messas

**Affiliations:** ^1^INSERM U1273, ESPCI Paris, CNRS FRE 2031, Physics for Medicine Paris, PSL Research University, Paris, France; ^2^Centre de Référence des Maladies Vasculaires Rares, Hôpital Européen Georges-Pompidou, Assistance Publique – Hôpitaux de Paris (APHP), Paris, France; ^3^INSERM U970 PARCC, Paris Descartes University – Sorbonne Paris Cité University, Paris, France; ^4^Service d’Anatomie Pathologique, Hôpital Européen Georges-Pompidou, Assistance Publique – Hôpitaux de Paris (APHP), Paris, France; ^5^Service de Radiologie Cardiovasculaire, Hôpital Européen Georges-Pompidou, Assistance Publique – Hôpitaux de Paris (APHP), Paris, France

**Keywords:** bicuspid aortic valve, thoracic aorta, arterial stiffness, Mucoid Extracellular Matrix Accumulation, ultrasound diagnosis, carotid artery disease

## Abstract

**Purpose of the Review:**

Bicuspid aortic valve (BAV) is associated with a significant risk of development of aneurysm and dissection of the ascending thoracic aorta. Development of what is called BAV associated aortopathy is particularly heterogeneous with an uncertain prognosis and with no prognostic biomarkers except for the aortic diameter. This situation leads to an important variability of the therapeutic strategy of this aortopathy. By reviewing the literature on aortic stiffness in the case of BAV, we aimed at evaluating its potential prognostic role in the development of aortic dilatation.

**Recent Findings:**

Studies evaluating aortic stiffness, with ultrasound or magnetic resonance imaging, converge toward the description of an increased segmental aortic stiffness in BAV patients regardless of age, diameter or aortic level, from the root to the arch. Even though there is a lack of longitudinal studies evaluating the progression of aortic dilatation, new data have recently shown the potential prognostic role of the maximal rate of systolic distension of the aortic wall with magnetic resonance imaging.

**Summary:**

Although the use of aortic distensibility calculation is a simple evaluation of stiffness that could be easily transposed in daily practice, its interpretation remains uncertain. New arterial stiffening indicators seem more promising but need a stronger validation.

## Introduction

Even though Bicuspid Aortic Valve (BAV) is the most common cardiac malformation with a prevalence estimated between 0.5 and 2% of the general population (Ward, 2000; Hoffman and Kaplan, 2002), the aortic prognosis in the case of BAV is still debated and remains to be the main question not yet answered in the literature. Medical and surgical management of what is called BAV associated aortopathy (BAV-AA) remains elusive with important variations in the recommendations due to the difficulty to predict the risk of acute aortic events (Borger et al., 2004). BAV-AA is difficult to estimate due to different thresholds used to define aortic dilatation (diameter over 35 or 40 mm, or normalized ratio to reference values), and age of the study population. For example, prevalence of aortic dilatation >40 mm in the Olmsted County cohort was 15% for BAV patients of 32 ± 20 years (Michelena et al., 2008), and 9.8% in the Toronto cohort with a population age of 35 ± 16 years (Tzemos et al., 2008). After a 9-year-follow up or 10 ± 6-year-follow up, aortic dilatation rate raised to 49% in the Olmsted County cohort and to 20% in the Toronto cohort, respectively. Della Corte et al. even reported a rate of 83% of aortic dilatation, while using a normalized ratio > 1.1 to define dilated patients (Della Corte et al., 2007).

Impact of BAV-AA on morbidity and mortality is related to the risk of acute aortic events, including dissection and aneurysm rupture. In the case of BAV, a higher aortic dissection risk has been suggested by numerous pathological studies (Gore and Seiwert, 1952; Larson and Edwards, 1984; Roberts and Roberts, 1991). The exact prevalence of aortic dissection remains poorly defined, ranging from 1 to 13% of BAV among aortic dissections (Ward, 2000). Among young people, BAV is one of the most important risk factors, representing 9% of all aortic dissections (Januzzi et al., 2004). However, even though BAV patients may have a relative risk of dissection 9 times greater than patients with normal tricuspid aortic valve (TAV), results of the literature converge toward a very moderate absolute risk of acute aortic event. Incidence of aortic dissection in the case of BAV was indeed estimated to 1.5/100,000 patient-years by Michelena et al. (2011) and at 18/100,000 patient-years in a recent Chinese cohort by Li et al. (2017), much lower than the 2/100 patient-years in the case of Marfan syndrome (Groth et al., 2017).

In daily practice, the risk for aortic dissection in BAV is assessed by the measure of the aortic diameter, the only morphological marker used to take therapeutic decisions, according to the most recent guidelines (Erbel et al., 2014). Aortic diameter threshold for surgical intervention was determined at 55 mm in the absence of coarctation, high blood pressure, or suspected family form of aortic dissection.

Aortic dilatation is a recognized risk factor for aortic dissection (Davies et al., 2006), but it doesn’t account for everything. Indeed, among the aortic dissections reported by the IRAD registry (not restricted to BAV), 40% of dissections occurred with an aortic diameter below 50 mm (Pape et al., 2007). Moreover, Davies et al. (2007) showed that despite a higher rate of aortic growth in BAV patients compared to controls (0.19 vs. 0.13 cm/year, *p* = 0.01), the incidence of rupture and dissection were similar. Because of early changes in the extracellular matrix, the elastic properties, especially the stiffness, of the aortic wall appear to be modified early in the case of BAV. This review focuses on the ascending aortic biomechanical properties in BAV. We aimed at gathering the whole set of results on the study of the biomechanical properties of the aortic wall, in order to highlight the interest of stiffness assessment with the different imaging modalities available.

## Structural Changes

### Histology

**FIGURE 1:**
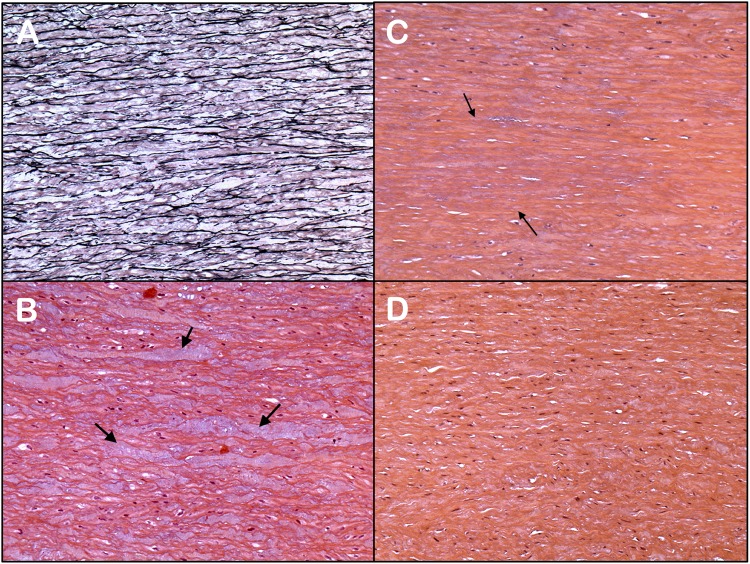
Different histological features observed in aortas of BAV patients **(A–C)** and the normal aorta **(D)**: **(A)** Fragmentation and loss of the elastin fibers (limited to 1 or 2 lamellar units; mild grade) (Elastic stain; Original X10). **(B)** Inter-lamellar degeneration with mucoid replacement (MEMA) (H&E stain; Original X10). **(C)** Moderate loss of nuclei of vascular smooth muscle cells (involving 4 to 10 lamellar units; area between arrows) (H&E stain; Original X10). **(D)** Normal ascending thoracic aorta. The media shows no mucoid accumulation in the extracellular matrix or loss of smooth muscle cells (H&E stain; Original X10).

Histological analyses of the ascending aortic wall of BAV patients have been widely described as non-specific abnormalities. They include loss of vascular smooth muscle cells (vSMC), loss and fragmentation of elastin fibers and/or Mucoid Extracellular Matrix Accumulation (MEMA) (Figure 1). They include loss of vSMC, loss and fragmentation of elastin fibers and/or MEMA, corresponding to an accumulation of mucoid substance (Figure 1; Halushka et al., 2016). MEMA corresponds to the former denomination of multiple terms, such as Erdheim’s cystic degeneration, cystic medial necrosis, medionecrosis, and cystonecrosis (Larson and Edwards, 1984; Bonderman et al., 1999; Bauer et al., 2002). Even though these non-specific abnormalities are present in a wide range of aortic pathologies, in the case of BAV patients, they occur at an early stage in the absence of aortic dilatation (Bonderman et al., 1999). There are, however, a marked heterogeneity of lesions observed in BAV patients (De Sa et al., 1999; Parai et al., 1999; Bauer et al., 2002; Leone et al., 2012; Waters et al., 2017). The pathophysiological process leading to the medial layer remodeling in the case of BAV is poorly understood. Two assumptions have been formulated and are not necessarily conflicting: on the one hand, a constitutive aortic wall alteration due to genetic predisposition, on the other hand an acquired aortic wall alteration due to the modified amplitude and direction of the flow wall shear stress, generated by the BAV (Girdauskas et al., 2011; Sievers and Sievers, 2011; Piatti et al., 2017; Liu et al., 2018). The presence of matrix degeneration is thus emphasized in areas with high shear stress (Gore and Seiwert, 1952; Gosline et al., 2002).

Elastic fragmentation and loss, vSMC loss, and MEMA are also found in a great proportion of other aortopathies, such as Marfan or Loeys-Dietz syndrome (Davies et al., 2007). The comparison between BAV and Marfan syndrome is justified due to common histological features affecting elastin fibers, vSMC, and due to an increased risk of aortic dissection in both pathologies. Grewal et al. (2016) underlined the common features of aortic wall involvement, with an alteration of fibrillin 1 expression and a lower differentiation of vSMC. However, in case of Marfan syndrome, histological abnormalities of the medial layer are diffuse and more severe (Della Corte et al., 2006; Waters et al., 2017). Marfan syndrome is clinically characterized by an earlier aortic dilation and a major risk of acute aortic event, much higher than in BAV (Detaint et al., 2014). Lastly, the presence of elastin fibers loss, vSMC loss, and MEMA, in BAV patients proves to be a non-specific marker of aortic fragility, without prejudging the causal genetic or haemodynamic origin.

### Immunohistochemistry

Immunohistochemical studies confirm the early loss of elastin fibers. By analyzing aortic aneurysms of BAV patients, Nataatmadja (2003) found a reduction of fibrillin, fibronectin, and tenascin in the extracellular matrix (Fedak et al., 2003; Nataatmadja, 2003). An overall content reduction of elastin fibers was also observed by Blunder et al. (2012). Changes in extracellular matrix content may be associated with either a modification of its synthesis or an increased expression of matrix metalloproteases (MMP). In the case of BAV, studies converge toward an increase in pro-MMP-2 expression in the ascending aorta, without modification of the MMP-9 level (Fedak et al., 2003; Lemaire et al., 2005). Moreover, the activity of MMP-2 seems to be correlated with the presence of important haemodynamic wall stress (Guzzardi et al., 2015).

### Micro-Architectural Analysis

Beyond the analysis by optical microscopy, few studies evaluated the aortic wall of BAV patients using electron microscopic analyses. Niwa et al. (2001) presented some analysis of a few BAV patients’ aorta. Their results are consistent with light microscopy, showing heterogeneous elastin fibers fragmentation and a further increase in collagen (Niwa et al., 2001). At the cellular level, Maleki et al. (2016) used electron microscopy to evaluate intimal and medial cells in the case of BAV, and found cell abnormalities evoking a defect of cellular maturation. In addition to the fine cell evaluation, composition and organization of the extracellular matrix can be assessed with advanced techniques of multiphoton microscopy. Results converge toward a different orientation of elastin and collagen fibers in the case of BAV, proof of the early parietal remodeling (Phillippi et al., 2014). A decrease in elastin fibers radial orientation was found specifically in regions were aorta was dilated in BAV patients (Tsamis et al., 2016). In dilated aortas from BAV patients vs. TAV patients, the elastin fibers architecture was more disrupted when the collagen beams still remained organized.

All the histological changes presented converge toward an early non-inflammatory modification of the aortic wall architecture in BAV. As changes in elastic and collagen bundles are associated with a modified biomechanical behavior of the aorta (Roach and Burton, 1957), the second part of this review focuses on the results of aortic stiffness studies.

## Stiffness Evaluation of the Aorta of Bav Patients

The histological alterations observed overlap with a qualitative and quantitative early alterations of the extracellular matrix. At a physiological pressure level, elastin fibers are very distensible (Wolinsky and Glagov, 1964), with an elastic modulus of 1.1 MPa for a stress below 1 MPa (Gosline et al., 2002). BAV histological features include MEMA, elastin fiber fragmentation, loss of smooth muscle cells, and augmentation of collagen fibers (de Sa et al., 1999). Longobardo et al. (2017) found an increase of elastin degradation, evaluated by the measurement of elastin soluble fragments, in the case of BAV associated with an increased aortic stiffness. The search for early markers of abnormalities of the thoracic aorta might be helpful to guide the management of the patients carrying BAV before the appearance of an aortic dilatation. In order to assess the aortic elastic properties, the local Young’s modulus (E) can be calculated, either invasively on a test-bed or non-invasively with two major indicators: distensibility and pulse wave velocity (PWV) following these formulas:

$$\text{E}=\frac{\text{dD}}{\text{h.Dist}};\text{E}=\frac{\text{ρ.dD}{\text{.PWV}}^{2}}{\text{h}}$$

E: Young’s modulus (Pa); dD: diastolic aortic diameter (m); h: wall thickness (m); Dist: distensibility (Pa^−1^); ρ: wall viscosity (Pa.s); PWV: pulse wave velocity (m.s^−1^).

### *Ex vivo* Mechanical Testing

The simplest evaluation of the local Young’s modulus of a tissue is to measure directly the elongation of an *ex vivo* portion by controlling the stress, as developed by Roach and Burton (1957). Pham et al. (2013) compared biomechanical properties by load-controlled biaxial testing of aorta specimens from patients with BAV to patients without BAV, who had all been operated for an ascending aortic aneurism. Mean aortic diameter [50.1 mm (BAV) vs. 49.4 mm (non-BAV)] and age [55.0 years (BAV) vs. 59.5 (non-BAV)] did not differ. Aortic specimens of BAV patients were significantly stiffer in the longitudinal direction at 60 N.m^−1^ membrane tension, and a similar trend, although non significant, was observed at 120 N.m^−1^ and in the circumferential direction. Histological analyses of the specimens retrieved features that are usually found in aortic wall in the case of BAV, including a lower proportion of elastin fibers. The increased aortic stiffness in BAV patients is in agreement with previous studies with different testing conditions (Choudhury et al., 2009; Duprey et al., 2010).

**FIGURE 2:**
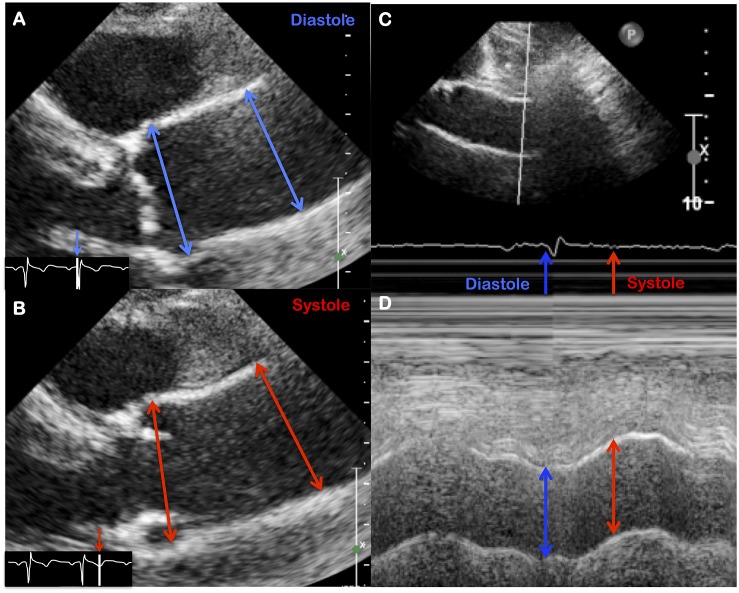
Evaluation of aortic strain using echocardiographic features, either with B mode **(A,B)** or with M mode **(C,D)**.

A higher macroscopic strength of aortic specimens is also found in BAV patients. Forsell et al. (2014) compared biomechanical properties by displacement-controlled uniaxial testing of aorta specimens from patients with BAV to patients without BAV, who had all been operated for an ascending aortic aneurism. Mean aortic diameters [59 mm (BAV) vs. 53 mm (non-BAV)] and ages [60 years (BAV) vs. 59 (non-BAV)] did not differ. Aortic strength of BAV patients was two times stronger (0.99 ± 0.46 vs. 0.49 ± 0.21 MPa; *p* < 0.001; Forsell et al., 2014). This difference was primarily explained by higher collagen stiffness in BAV patients. The increased aortic strength in BAV patients is in agreement with previous studies (Okamoto et al., 2002; Pichamuthu et al., 2013).

Although these *in vitro* tests do not have an extension in clinical practice, they highlight the increase in aortic stiffness and strength under fixed conditions at a tissue length scale and show the challenge of an accurate evaluation at the organ length scale.

### Ultrasonic Stiffness Evaluation

Evaluation of the ascending aorta by trans-thoracic ultrasound seems the simplest, most widely used and low-cost approach. Aortic distensibility and stiffness index can be easily calculated with time-motion (M-mode) or brightness (B-mode) acquisitions (Figure 2), as follows (Hirai et al., 1989; Nistri et al., 2008):

$$\text{Dist}=2\frac{\text{sD}-\text{dD}}{\text{SBP}-\text{DBP}};\text{SI}=\frac{\text{Ln }\left(\frac{\text{SBP}}{\text{DBP}}\right)}{\left(\frac{\text{sD}-\text{dD}}{\text{dD}}\right)}$$

Dist: distensibility (mmHg^−1^); sD: systolic aortic diameter (mm); dD: diastolic aortic diameter (mm); SBP: systolic blood pressure (mmHg); DBP: diastolic blood pressure (mmHg); SI: stiffness index (no unit).

The wall stress quantification is only evaluated by the measurement of brachial blood pressure. Due to the restricted field of view, the evaluation is limited to the first centimeters of the ascending aorta. Despite these limitations, evaluation of aortic distensibility allowed a wide screening of BAV patients and can be performed during the usual follow-up of these patients. Thus, ultrasonic data represent the core data of stiffness evaluation in BAV associated aortopathy with distensibility analysis in numerous clinical series.

**Table 1:** Results of ultrasonic stiffness evaluation (stiffness index, SI) at the tubular aortic level.

Reference	BAV				Control			
	*N*	Age (years)	Tubular aorta (mm)	Aortic SI (No. unit)	*N*	Age (years)	Tubular aorta (mm)	Aortic SI (no unit)
Oulego-Erroz et al., 2013	24	6.5 ± 5.0	20.1	7.2 ± 4.5	24	7.0 ± 5.0	16.3 ± 3.5	4.1 ± 2.3
Weismann et al., 2016	50	10.9 (0.2–20.2)	Not provided	3.5 (1.4–7.5)	50	11 (0.3–17.9)	Not provided	2.8 (1.5–4.6)
Santarpia et al., 2012	40	20.9 ± 4.7	30 ± 0.5	6.4 ± 3.5	40	23.4 ± 3.4	2.6 ± 0.3	3.9 ± 1.2
Nistri et al., 2008	127	23 ± 10	29.9 ± 5.3	7.5 ± 5.0	114	21 ± 10	23.8 ± 4.4	3.6 ± 1.89
Drapisz et al., 2013	20	27 (24–33)	31 (28–38)	8.9 (6.5–10.1)	20	30 (25–33)	26 (24–30)	3.7 (2.5–4.6)
Schaefer et al., 2007	29 (1LR)	43 (1LR)	32.2 ± 5.1 (1LR)	6.4 (1LR)	–	–	–	–
	29 (1NR)	39 (1NR)	31.8 ± 6.4 (1NR)	9.6 (1NR)
				
Li et al., 2016	28 (non-dilated),	41	28.2 ± 4.9,	8.3 ± 4.9,	29	42 ± 11	26.4 ± 3.5	5.2 ± 2.7
	29 (dilated)	49	41.0 ± 5.3	19.7 ± 14.1
Goudot et al., 2018a	108	48.8 ± 16.6	39.7 ± 8.6	20.4 ± 31.3	148	45.6 ± 17.4	30.2 ± 5.4	12.7 ± 14.8

*Reference order is presented depending on the age of the population studied.*

Overall, ultrasonic evaluation studies converge toward an increase in aortic stiffness and a larger aortic diameter, concordant with *ex vivo* studies (Schaefer et al., 2007; Nistri et al., 2008; Santarpia et al., 2012; Oulego-Erroz et al., 2013; Li et al., 2016). Aortic stiffness increases early, independently of the valvular function and of the initial dilatation level. Three series evaluated young BAV patients (mean age of 6.5 years in Oulego-Erroz et al., 2013 and 8.3 years in Pees and Michel-behnke, 2012 and 10.9 years in Weismann et al., 2016) and revealed a 62, 29, and 25% increase in aortic stiffness, respectively, as compared to controls, in the absence of aortic dilatation (Pees and Michel-behnke, 2012; Oulego-Erroz et al., 2013; Weismann et al., 2016). These studies on young patients emphasize the early increase in stiffness prior to the onset of aortic dilation. This aortic stiffness increase persists in adulthood, as demonstrated by Nistri et al. (2008) with a young adult population of 127 patients with an average age of 23 years, and is then maintained throughout life, confirmed by studies of older populations which were compared to TAV controls in each case (Santarpia et al., 2012; de Wit et al., 2013; Drapisz et al., 2013; Lee et al., 2015; Li et al., 2016). In those studies, the influence of the function or the morphotype of the valve seemed limited. Schaefer et al. (2007), however, found that BAV with 1-LR morphotype was associated with a stiffer aorta, compared to 1-NR type, but only at the sinus of Valsalva level (Schaefer et al., 2007). Ultrasound evaluation can also provide an assessment of segment circumferential stiffness along with the aortic axis (sinus of Valsalva, tubular aorta, aortic arch). This approach may have a particular interest as BAV associated aortopathy presents a segmental histological injury. We recently evaluated the segmental stiffness of the ascending aorta of BAV patients. Although all aortic segments had a higher stiffness in the case of BAV, only the sinus of Valsalva remained more stuff, regardless of the aortic dilatation (Goudot et al., 2018a). These results are consistent with the previous data on an intrinsic involvement of the sinus of Valsalva in BAV (Azadani et al., 2012; Yassine et al., 2017) and highlight the need to distinguish stiffness according to the different aortic segments.

The hypothesis of a genetic background leading to the formation of a BAV and the associated aortopathy is supported by study results on syndromic forms of BAV (Turner-, Williams-Beuren-, Shone-, DiGeorge- syndromes). They represent a distinct group of BAV patients, with a high prevalence of other associated cardiac and aortic abnormalities, including ventricular septal defect and aortic coarctation. In these patients, the genetic background of BAV and cardiac or aortic defect is clear. Assessment of aortic distensibility in a series of women with Turner syndrome, particularly affected by acute aortic events (Matura et al., 2007), revealed an early increase in their ascending aorta stiffness (De Groote et al., 2017).

To our knowledge, no prognostic study has yet stated the aortic stiffness index as a predictive marker of further aortic dilatation. Although stiffness deserves attention because it precedes aortic dilatation, ultrasonic evaluation (either with M or B-mode) suffers from a great variability of values, between the BAV and TAV patient groups (Table 1), but also from poorly standardized measurements, operator dependency, and the need for coupling with an accurate measurement of central pressure. For these reasons, other morphological markers of aortic stiffness, with a minor variability depending on the operator, are required. For example, aortic wall longitudinal strain measurement with speckle tracking imaging may be a promising technique. Aortic speckle tracking was developed to obtain the longitudinal and circumferential deformation of the aortic wall (Oishi et al., 2008). With the advantage of a simple implementation, only based on cardiac ultrasound loops, 2D evaluation remains the main limitation, although 3D is under development (Karatolios et al., 2013). In its 2D use, however, absolute mean difference ± SD between longitudinal strain measurements, within one observer and between two observers, was low at 1.5 and 1.1%, respectively (Longobardo et al., 2017).

### Stiffness Assessment With Magnetic Resonance Imaging (MRI)

The development of MRI in cardiovascular pathologies now provides a powerful tool to assess the stiffness of the entire thoracic aorta, not only with the local evaluation, based on distensibility, but also the regional stiffness with the PWV measurement.

Following ultrasound studies, aortic distensibility using MRI has been initially investigated by Grotenhuis et al. (2007) at the level of the sinotubular junction with 2D sequences. In 20 BAV patients as compared with 20 matched controls, a reduced root distensibility (3.1 ± 1.2.10-3 mmHg^−1^ vs. 5.6 ± 3.2.10-3 mmHg^−1^, *p* < 0.01) was consistent with the higher concomitant PWV measures (5.6 ± 1.3 m/s vs. 4.5 ± 1.1 m/s, *p* < 0.01), converging toward a higher aortic stiffness in the case of BAV (Grotenhuis et al., 2007).

This observation was confirmed by Burris et al. in 65 young BAV patients (mean age of 28 years) with a tendency for a higher PWV (6.53 vs. 3.51 m/s, *p* = 0.1) but with a large variation among BAV patients (SD of 5.88 m/s). More recently, the PWV can be measured from the 4D flow MRI sequences, allowing a reconstruction of the entire aortic arch (Guala et al., 2018). However, there are several limitations to measuring PWV with MRI: sequences require a long acquisition time (10 min of total scan time Guala et al., 2018), and are generally post-processed afterward. Lastly, it is a flow evaluation, without direct evaluation of the behavior of the aortic wall.

Pulse wave velocity appears as a complex biomarker, as it can be measured with very different approaches (Time to foot, time to peak upslope, Fourier analysis, cross correlation or center of mass method). Thus, variable results could be obtained in healthy adults, depending on which part of the waveform is analyzed, as reported by Dyverfeldt et al. (2014). For example, in the Bland–Altman analysis, the bias was 2.21 ± 1.1 m/s between the cross correlation and the time to foot method.

To better differentiate TAV and BAV patients, new biomarkers of aortic stiffening based on aortic strain analysis over the cardiac cycle have been proposed by Aquaro et al. (2011). In this study, 2 indexes, the Maximal Rate of Systolic Distension (MRSD) and the Maximal Rate of Diastolic Recoil (MRDR) have been tested in a BAV population. These measurements are accessible from a cross-section made on the ascending tubular aorta, perpendicular to the axis of the vessel. The circumferential aortic wall was tracked continuously over the cardiac cycle during a single acquisition with a temporal resolution of about 1 ms. They correspond to the dynamic change of the aortic diameters. However, a limitation of these indicators is the transverse evaluation, that may be affected by longitudinal movements of the aorta during the cardiac cycle. Both of these new biomarkers have proven to be much lower in BAV-patients than in controls, even in the absence of thoracic aortic dilatation. Moreover, receiver operating characteristic curve analysis of MRSD distinguished BAV from controls with 100% sensitivity and 95% specificity. In a longitudinal study with a short follow-up of 17 months, the same authors showed that MRSD could predict an increase of aortic diameter with 93.7% specificity and 75.6% sensitivity, with a cut-off value of MRSD ≤ 6 (Aquaro et al., 2017).

### Global Evaluation of Aortic Stiffness: Carotid-Femoral PWV (cfPWV)

Apart from the usual imaging techniques of the ascending aorta, PWV has been first developed and validated with non-invasive measures of the pulse wave at two sites of the arterial tree. Carotid-femoral PWV, calculated by applanation tonometry gives access to a global aortic stiffness. Although this technology is not widespread in daily practice, a large validation of its use and prognosis relevance has already been achieved in non-BAV patients (Laurent et al., 2001; Reference Values for Arterial Stiffness’ Collaboration, 2010). Measurement of the cfPWV has the disadvantage of an average stiffness evaluation over the whole aorta. In case of BAV, only the ascending thoracic aorta seems to be affected by structural changes. Results of the carotid-femoral PWV should thus be interpreted with caution, considering that it is an average stiffness including regions of unequal stiffness.

Tzemos et al. (2010) showed a moderate increase in regional stiffness using cfPWV in a BAV population with aortic dilatation: 9.3 (9.0–10.0) cm.s^−1^ in BAV (mean age 31 years, *n* = 16) vs. 7.0 (6.9–7.2) cm.s^−1^ in controls. Results in BAV patients with non-dilated aorta were similar to controls with a mean PWV of 7.0 (6.9–7.4) cm.s^−1^. Moreover Warner et al. and Shim et al. did not find any difference of cfPWV between non-dilated BAV patients and controls (Grotenhuis et al., 2007; Shim et al., 2011). Compared to the increased cfPWV demonstrated in Marfan syndrome (Salvi et al., 2018), these results converge toward a more localized alteration of the elastin fibers among large vessels. However, interpretation of cfPWV in BAV patients has to be taken with caution due to the limited number of patients included (16 and 10 BAV patients, respectively).

## Clinical Relevance of Aortic Stiffness Indicators

Arterial wall with increased stiffness may be due to an extracellular matrix remodeling, mainly driven by a decrease in the proportion of elastin fibers, as presented above. A stiffer aorta presents a lower deformation capacity when facing the different sources of stress (longitudinal, axial, circumferential stress), and therefore presents a higher risk of dissection or rupture. Even though, from a biomechanical point of view, arterial rupture appears when blood pressure exceeds the intrinsic wall resistance, the prognostic impact of stiffness evaluation in BAV faces many inconsistencies related to the ill-defined pathophysiology of acute aortic events. The rate of acute aortic events in BAV patients is indeed lower than their increased aortic stiffness would suggest. Among BAV patients, imaging studies and *in vitro* studies of the aortic properties often emphasized the segmental and particularly heterogeneous characteristics of BAV associated aortopathy. As a consequence, only a small but highly impacted aortic wall segment with altered matrix could be the starting point of a dissection, which then spreads to the entire aorta.

Thus, only prospective studies evaluating stiffness indicators on the subsequent development of aortic aneurysm and acute aortic events could validate these biomarkers as prognostic ones. There is currently very limited data on this topic. Only MRSD, measured with MRI and recently validated by Aquaro et al. (2017) as an aortic dilatation marker, gave a prognostic value among BAV patients (Nollen et al., 2004; Aquaro et al., 2017).

However, designing a follow-up study to evaluate predictive risk markers of aortic dissection in BAV is complex and uncertain due to the unexpected low incidence of aortic dissection in BAV patients. Moreover, it would need inclusion of numerous BAV patients as homogeneous as possible. The development of regional stiffness indicators in association with a better evaluation of the wall stress will also allow a better characterization of the different BAV forms.

## Analysis of Arterial Stiffening: Toward New Tissue Biomarkers of Aortic Stiffening?

The main objective of this review was to stimulate future directions for the evaluation of aortopathy associated with BAV. Methods for assessing arterial stiffness (mainly distensibility and PWV) showed a stiffness difference between BAV and normal patients at the thoracic aorta level. However, analysis of the raw data showed a significant overlap of the stiffness values, and a significant amount of variability in the measurements with conventional imaging without any truly prognostic role. Interestingly, the MRSD, recently validated as a prognostic tool, is more than a simple stiffness indicator. The peak of distensibility variation appears indeed to be independent on the blood pressure, and to correspond to the dynamic aortic behavior when pressure rises. Switching from the evaluation of the arterial stiffness to the evaluation of the arterial stiffening over the cardiac may bring a new insight into the inner wall properties, as suggested by recent studies (Mirault et al., 2015; Gavish and Izzo, 2016; Goudot et al., 2018b). Arterial stiffness, strongly influenced by the blood pressure level, undergoes a continuous variation during the cardiac cycle. The arterial stiffening is a physiological condition allowing a constant adaptation of the artery to different levels of stress, regardless of its initial stiffness. This parameter is thereby less dependent on the blood pressure level.

In an evaluation of carotid arterial stiffening on patients suffering from vascular Ehlers–Danlos syndrome (vEDS), responsible for arterial ruptures due to defectuous collagen type III in the arterial wall matrix, Mirault et al. demonstrated that basal carotid stiffness was not different in vEDS patients vs. controls, whereas the stiffening over the cardiac cycle was reduced in the vEDS group (Mirault et al., 2015). In this study, the use of ultrafast ultrasonic imaging, evaluating different components of the pulse wave at different times during the cardiac cycle, could measure the arterial stiffening over the cardiac cycle, opening the field to new non-invasive parameters. However, there are currently very few tools to evaluate the arterial stiffening in daily practice. Even though new arterial stiffening biomarkers appear to be a promising tool in aortic wall assessment, the development of dynamic imaging with both MRI and ultrasound imaging requires more validation among the most achievable homogeneity in BAV-patient cohorts.

## Conclusion

Regardless of age, presence of valvular dysfunction, sporadic, genetic or syndromic forms of BAV patients, early and segmental changes in the aortic wall result in an excess of wall stiffness as compared to patients with a normal TAV. These results are consistent with the histological aspect of BAV associated aortopathy with marked lesions of elastin fibers destruction and mucoid material replacement. Aortic stiffness markers require a better reliability and long-term evaluation to conclude on their useful prognostic roles. Standardization of stiffness parameters, coupling to flow data, and perhaps evaluation of aortic stiffening could allow clinical parameters to be developed in the near future for prognostic purposes on the risk of dilatation and possibly acute aortic event.

## Author Contributions

GG and TM wrote the manuscript. PB proofread the histology part of the manuscript. GS proofread the part of the manuscript concerning the evaluation of the aorta by magnetic resonance imaging. MP proofread the part of the manuscript concerning the biomechanical properties of the aorta as well as the ultrasound evaluation. EM performed the final approval of the version to be published and agreed to be accountable for all aspects of the work in ensuring that questions related to the accuracy and integrity of any part of the work are appropriately investigated and resolved.

## Conflict of Interest Statement

The authors declare that the research was conducted in the absence of any commercial or financial relationships that could be construed as a potential conflict of interest.
